# Development of a New Surface Acoustic Wave Based Gyroscope on a X-112°Y LiTaO_3_ Substrate

**DOI:** 10.3390/s111110894

**Published:** 2011-11-21

**Authors:** Wen Wang, Jiuling Liu, Xiao Xie, Minghua Liu, Shitang He

**Affiliations:** State Key Laboratory of Acoustics, Institute of Acoustics, Chinese Academy of Sciences, Beijing 100190, China; E-Mails: liujiuling@mail.ioa.ac.cn (J.L.); xiexiao08@mails.gucas.ac.cn (X.X.); liuminghua@mail.ioa.ac.cn (M.L.); heshitang@mail.ioa.ac.cn (S.H.)

**Keywords:** Coriolis force, gyroscopic effect, SAW gyroscope, surface effective permittivity

## Abstract

A new micro gyroscope based on the surface acoustic wave (SAW) gyroscopic effect was developed. The SAW gyroscopic effect is investigated by applying the surface effective permittivity method in the regime of small ratios of the rotation velocity and the frequency of the SAW. The theoretical analysis indicates that the larger velocity shift was observed from the rotated X-112°Y LiTaO_3_ substrate. Then, two SAW delay lines with reverse direction and an operation frequency of 160 MHz are fabricated on a same X-112°Y LiTaO_3_ chip as the feedback of two SAW oscillators, which act as the sensor element. The single-phase unidirectional transducer (SPUDT) and combed transducers were used to structure the delay lines to improve the frequency stability of the oscillator. The rotation of a piezoelectric medium gives rise to a shift of the propagation velocity of SAW due to the Coriolis force, resulting in the frequency shift of the SAW device, and hence, the evaluation of the sensor performance. Meanwhile, the differential structure was performed to double the sensitivity and compensate for the temperature effects. Using a precise rate table, the performance of the fabricated SAW gyroscope was evaluated experimentally. A sensitivity of 1.332 Hz deg^−1^ s at angular rates of up to 1,000 deg s^−1^ and good linearity are observed.

## 1. Introduction

Recently, the surface acoustic wave (SAW) based gyroscope has provided a new method for angular rate detection with excellent inherent shock robustness, very larger dynamic testing range, small size, low cost, simplicity and long working life [1]. A typical SAW gyroscope consists of a two-port SAW resonator to generate a stable standing wave and a SAW delay line pattern to detect the second SAW induced by Coriolis force acting on the metallic masses distributed along the anti-node position of the standing wave. Some groups have reported such SAW-based gyroscopes with different designs and structures. Jose *et al.* first reported a successful SAW gyroscope configuration based on a standing-wave mode with a voltage sensitivity of 2.67 mV s deg^−1^ [2]. Varadan *et al.* presented the design and performance evaluation of a 74.2 MHz microelectromechanical system-interdigital transducer (MEMS-IDT) SAW gyroscope with a similar structure [3]. Zhang and Wang present an optimal design of the SAW device using the coupling of modes, considering the effect of metallic dot thickness on sensor performance [4]. However, despite some reported important works on such SAW gyroscopes, they still suffer from low precision (submicron voltage detection) and poor temperature stability due to the use of large piezoelectric-coupling substrate materials like LiNbO_3_ with high temperature coefficients.

To overcome the shortcomings of SAW gyroscopes based on the standing wave mode, another gyroscope mode was reported utilizing the SAW gyroscopic effect, which originates from the rotation effect of a wave that is a rotation vector perpendicular to the propagating axis, causing a velocity change proportional to the input rotation through the Coriolis force [5]. There are now several dozens of papers that analyze the SAW gyroscopic effect. Destrade *et al.* studied the propagation of SAW over a rotating orthorhombic crystal [6]. Biryukov *et al.* described the SAW gyroscopic effects by solving the piezoelectric medium equations of motion [7]. A shift of SAW velocity due to external rotation was determined for various piezoelectric substrates. These papers are oriented mostly on the complex procedure of a dispersion equation derivation in an explicit form to find then its numerical solution. To simply the theoretical calculation, a perturbation theory was introduced to the gyroscopic effect analysis, and some meaningful results were reported [8]. However, due to the neglect of the piezoelectricity of the rotated medium and only consideration of the first order in perturbation series, the calculated results deviated obviously from the experimental values [8].

Until recent years, the theoretical work on gyroscopic effect was not confirmed by the experiments. Lee *et al.* first realized a prototype of a micro rate gyroscope based on the SAW gyroscopic effect on ST quartz using the differential dual-delay-line oscillator configuration [8,9], temperature compensation was also conducted satisfactory. However, a very low frequency sensitivity of 0.431 Hz deg^−1^ s was observed at angular rates up to 2,000 deg/s, and this is far away from any real application. To improve the detection sensitivity, some other meaningful research works on such gyroscope were also done [10,11].

The first purpose of this study is to establish a comprehensive theoretical model dealing with the SAW based gyroscopic effect. A surface effective permittivity method based on the function of acoustic waves in piezoelectric materials and the boundary conditions was introduced to the gyroscopic effect analysis [12], which provides a simple way to describe the SAW propagating velocity shift caused by external rotation, the gyroscopic gain constant characterizing the performance of the gyroscope and expected sensitivity were also depicted in detail. Moreover, the gyroscopic effect of SAW propagating along various piezoelectric substrates was analyzed, and the calculated results indicate that excellent sensor response will be observed from a rotated X-112°Y LiTaO_3_ piezoelectric substrate.

**Figure 1.**
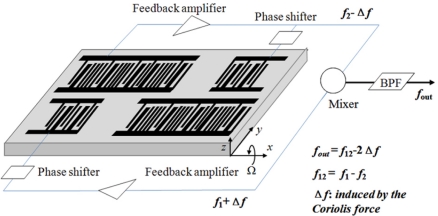
The schematic of the present SAW gyroscope.

The second aim of this study is to develop a valuable SAW gyroscope based on an X-112°Y LiTaO_3_ piezoelectric substrate. It was composed of a dual-delay line oscillator, in which, two parallel delay lines with opposite propagation direction wer fabricated on the same chip as the feedback element. The schematic of the sensor is shown in Figure 1.

When the device was subjected to rotation around the x-axis, Ω_x_, the Coriolis force acts on the particles along the SAW propagation path, the induced pseudo-SAW couples with the initial SAW, resulting in the SAW velocity change. Then the SAW velocity in one delay line increases and that of the other one decreases due to the opposite rotation around the x-axis. Hence, the differential scheme doubles the sensitivity of the sensor and compensates for the temperature effect, as indicated in Figure 1. The input rotation was characterized by the differential oscillation frequency *f*_out_. To ensure the excellent frequency stability, the single phase unidirectional transducers (SPUDT) and combed transducers were used to structure the SAW device [13–15]. Using a precise rate table, the sensor performance of the fabricated gyroscope with operation frequency of 160 MHz was evaluated. High sensitivity and good linearity were observed.

## 2. Theoretical Analysis of the SAW Gyroscopic Effect

### 2.1. SAW Gyroscopic Effect

**Figure 2.**
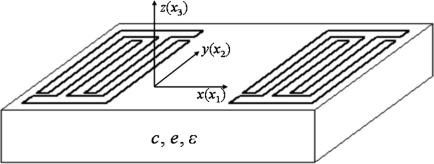
The coordinate system used in this study.

A plane SAW is propagated along the surface of an anisotropic piezoelectric substrate occupying the half space as shown in Figure 2. The wave propagates along the *x*-axis. If the substrate rotates around the *x*-axis with a constant angular rate, particles of the wave are subjected to additional inertial forces, including the Coriolis and centrifugal forces. With the counter-clockwise rotation around the *x*-axis, the Coriolis force acts on the vibrating particles and induces a pseudo running wave shifted by a quarter of a wavelength, and coupling with the initial SAW, resulting in the change of trajectory of the wave particles, and hence, the acoustic wave displacement was deviated, leading to the acoustic wave velocity shift. Consequently, a frequency variation proportional to the input rotation according to the relationship among the frequency was expected. The theoretical analysis of the SAW gyroscopic effect over the whole depth is given in the following section.

### 2.2. Theoretical Model

In this section, the SAW gyroscopic effect is described by solving the piezoelectric medium equations of motion and surface effective permittivity method. Consider an anisotropic and piezoelectric medium occupying a half-space (*x*_3_ ≤ 0) with no mechanical load about the plane (*x*_3_ = 0) and rotating at a constant angular rate (Ω*_i_*) about the *x*_i_-axis (*i* = 1,2,3), as schematically illustrated in Figure 2. The dynamic equations of linear piezoelectricity with Coriolis and centrifugal forces contribution take the following form in this coordinate system mentioned in Figure 1:

(1) $$\{\begin{array}{l} \rho \partial^{2}u_{i}/{\partial t}^{2}-C_{\mathrm{ijk}l}\partial^{2}u_{k}/\partial x_{j}\partial x_{l}-e_{\mathrm{kij}}\frac{\partial^{2}\phi }{\partial x_{k}\partial x_{j}}+2\rho E_{\mathrm{ijk}}\Omega_{j}\frac{\partial u_{k}}{\partial t}\\ \begin{array}{cc} +\rho \left(\Omega_{i}\Omega_{j}u_{j}-\Omega_{j}^{2}u_{i}\right)=0, & i,j,k,l=1,2,3 \end{array}\\ e_{\mathrm{jkl}}\frac{\partial^{2}u_{k}}{\partial x_{j}\partial x_{l}}-\varepsilon_{\mathrm{jk}}\frac{\partial^{2}\phi }{\partial x_{j}\partial x_{k}}=0 \end{array}$$

where *x*_1_, *x*_2_, *x*_3_, denote the Cartesian coordinates *x*, *y*, *z* respectively, *E*_ijk_ is the Levi-civita symbol, and we denote by *u_i_* the mechanical displacements and by *ϕ* the electric potential. *c_ijkl_*, *e_kij_*, and *ε*_ij_ stand for the elastic, piezoelectric and dielectric constants, and ρ for the mass density of the substrate, respectively. The summation convention for repeated tensor indices and the convention that a comma followed by an index denotes partial differentiation with respect to the coordinate associated with the index are adopted. The indices *i*, *j*, *k*, and *l* range from 1 to 3. With the compressed matrix notation [16], the material constants *c_ijkl_* and *e_ijk_* in Equation (1) can be represented by matrices *c_pq_* and *e_ip_*, with the convention that *p*, *q* = 1, 2, 3, , , 6. Similarly, the strain tensor *S*_ij_ and the stress tensor *T_ij_* can be represented by *S_p_* and *T_q_*. The first three terms in the left-hand side of the Equation (1) are the equation of the motion related to the inertia, the elasticity and piezoelectricity. The fourth and the fifth terms are due to the Coriolis force and the centrifugal force, respectively.

First, we assume a general solution of the Equation (1), the particle displacement and electrical potential, in the form:

(2) $$\{\begin{array}{l} \begin{array}{cc} u_{i}=A_{i}\text{exp}\left\{jk_{s}\left(x_{1}+\alpha x_{3}\right)-j\omega t\right\}, & i=1,2,3; \end{array}\\ \varphi =A_{4}\text{exp}\left\{jk_{s}\left(x_{1}+\alpha x_{3}\right)-j\omega t\right\} \end{array}$$

where *k_s_* and *ω* are the wave number in the *x*_1_ direction and the time frequency, respectively. *α_s_* is a decay constant along the *x*_3_ direction. *A_j_* (*j* = 1, 2, 3) and *A*_4_ are wave amplitudes. Substitution of Equation (2) into Equation (1) leads to a four linear algebraic equations (Cristoffel equation) for *A_j_* and *A*_4_ as follows:

(3) $$\left[\begin{array}{cccc} \Gamma_{11}-\rho V_{s}^{2} & \Gamma_{12} & \Gamma_{13} & \Gamma_{14}\\ \Gamma_{12} & \Gamma_{22}-\rho V_{s}^{2} & \Gamma_{23} & \Gamma_{24}\\ \Gamma_{13} & \Gamma_{23} & \Gamma_{33}-\rho V_{s}^{2} & \Gamma_{34}\\ \Gamma_{14} & \Gamma_{24} & \Gamma_{34} & \Gamma_{44} \end{array}\right]\left[\begin{array}{c} A_{1}\\ A_{2}\\ A_{3}\\ A_{4} \end{array}\right]=0$$

where:

$$\begin{array}{l} \Gamma_{11}=c_{55}\alpha^{2}+2\alpha c_{15}+c_{11};\;\;\Gamma_{12}=c_{45}\alpha^{2}+\alpha \left(c_{14}+c_{56}\right)+c_{16}+2j\rho V_{s}^{2}\left(\Omega_{3}/\omega \right)\\ \Gamma_{13}=c_{35}\alpha^{2}+\alpha \left(c_{13}+c_{55}\right)+c_{15}-2j\rho V_{s}^{2}\left(\Omega_{2}/\omega \right)\\ \Gamma_{14}=e_{35}\alpha^{2}+\alpha \left(e_{15}+e_{31}\right)+e_{11};\;\;\Gamma_{21}=c_{45}\alpha^{2}+\alpha \left(c_{14}+c_{56}\right)+c_{16}-2j\rho V_{s}^{2}\left(\Omega_{3}/\omega \right)\\ \Gamma_{22}=c_{44}\alpha^{2}+2\alpha c_{46}+c_{66};\;\;\Gamma_{23}=c_{34}\alpha^{2}+\alpha \left(c_{36}+c_{45}\right)+c_{56}+2j\rho V_{s}^{2}\left(\Omega_{1}/\omega \right)\\ \Gamma_{24}=e_{34}\alpha^{2}+\alpha \left(e_{14}+e_{36}\right)+e_{16};\;\;\Gamma_{31}=c_{35}\alpha^{2}+\alpha \left(c_{13}+c_{55}\right)+c_{15}+2j\rho V_{s}^{2}\left(\Omega_{2}/\omega \right)\\ \Gamma_{32}=c_{34}\alpha^{2}+\alpha \left(c_{36}+c_{45}\right)+c_{56}-2j\rho V_{s}^{2}\left(\Omega_{1}/\omega \right)\\ \Gamma_{33}=c_{33}\alpha^{2}+2\alpha c_{35}+c_{55};\;\;\Gamma_{34}=e_{33}\alpha^{2}+\alpha \left(e_{13}+e_{35}\right)+e_{15}\\ \Gamma_{44}=-\varepsilon_{33}\alpha^{2}-2\alpha \varepsilon_{13}-\varepsilon_{11};\;\;\Gamma_{41}=\Gamma_{14};\Gamma_{42}=\Gamma_{24};\Gamma_{43}=\Gamma_{34} \end{array}$$

Then, for nontrivial solutions of *A_j_* and/or *A*_4_, the determinant of the coefficient matrix of the linear algebraic equations must vanish, and this leads to a polynomial equation of degree eight for *α_s_*. The coefficients of this polynomial equation are generally complex. To ensure the decrease in the displacement *u_i_* and the potential *ϕ* into the substrate, the generally complex constant *α_s_* must have a negative imaginary part. Thus, we select four eigenvectors with negative imaginary part denoted by *α_s_*(*m*), and the corresponding eigenvectors by *A_j_*(*m*), *A*_4_(*m*), *m* = 1, 2, 3, 4. Thus, the general wave solution to Equation (1) in the form of Equation (2) can be written as:

(4) $$\{\begin{array}{l} u_{i}=\sum_{m=1}^{4}C(m){A'}_{i}^{\left(m\right)}\text{exp}\left\{{\mathrm{jk}}_{s}\left(x_{1}+\alpha \left(m\right)x_{3}\right)-j\omega t\right\},\;\;\;i=1,2,3\\ \varphi =\sum_{m=1}^{4}C(m){A'}_{4}^{\left(m\right)}\text{exp}\left\{{\mathrm{jk}}_{s}\left(x_{1}+\alpha \left(m\right)x_{3}\right)-j\omega t\right\} \end{array}$$

where *C_m_*(*m = 1*, *2*, *3*, *4*) are the weight factors.

The solutions of the motion equation satisfy both the mechanical boundary condition, and the electrical boundary condition respectively.

The mechanical boundary condition:

(5) $$\begin{array}{cc} T_{i3}{\left(x_{1},x_{3}\right)|}_{x_{3}=0}=c_{i3kl}\frac{{\partial u}_{k}}{{\partial x}_{l}}+e_{\mathrm{ki}3}\frac{\partial \phi }{\partial x_{k}}=0 & i,k,l=1,2,3 \end{array}$$

where *T_i3_* is the stress on SAW propagating direction.

The electrical boundary conditions were considered in case of free surface and metallic surface.

For a free surface, the effect of the electric field in the surrounding space can be considered by requiring the electrical potential cross the interface continuously:

(6) $$\{\begin{array}{l} {\varphi^{\prime }|}_{x_{3}=0^{+}}={\varphi |}_{x_{3}=0^{-}}\\ {\varphi^{\prime }(x_{1},\;x_{3})|}_{x_{3}>0}=\varphi (x_{1},0)\text{exp}(-2\pi f|s|x_{3}) \end{array}$$

also, the surface charge density σ(*x*_1_) created by the interdigital transducers (IDT) electrodes equal to the incontinuity of the normal component *D*_3_ of the dielectric displacement vector cross the interface:

(7) $${D_{3}(x_{1},\;x_{3})|}_{x_{3}=0^{+}}-{D_{3}^{\prime }(x_{1},x_{3})|}_{x_{3}=0^{-}}=\sigma (x_{1})$$

And then, substituting Equation (4) into the boundary conditions Equations (5–7) yields the following four linear algebraic equations for *C*(*m*):

(8) $$\left[\begin{array}{cccc} r_{11} & r_{12} & r_{13} & r_{14}\\ r_{21} & r_{22} & r_{23} & r_{24}\\ r_{31} & r_{32} & r_{33} & r_{34}\\ r_{41} & r_{42} & r_{43} & r_{44} \end{array}\right]\left[\begin{array}{c} C_{1}\\ C_{2}\\ C_{3}\\ C_{4} \end{array}\right]=\left[\begin{array}{c} 0\\ 0\\ 0\\ -j\bar{\sigma }\left(s\right)/\left(2\pi fs\right) \end{array}\right]$$

where:

$$\{\begin{array}{l} r_{1n}=\left(c_{15}+c_{55}\alpha_{n}\right)A_{1}^{\left(n\right)}+\left(c_{56}+c_{45}\alpha_{n}\right)A_{2}^{\left(n\right)}+\left(c_{55}+c_{35}\alpha_{n}\right)A_{3}^{\left(n\right)}+\left(e_{15}+e_{35}\alpha_{n}\right)A_{4}^{\left(n\right)}\\ r_{2n}=\left(c_{14}+c_{45}\alpha_{n}\right)A_{1}^{\left(n\right)}+\left(c_{46}+c_{44}\alpha_{n}\right)A_{2}^{\left(n\right)}+\left(c_{45}+c_{34}\alpha_{n}\right)A_{3}^{\left(n\right)}+\left(e_{14}+e_{34}\alpha_{n}\right)A_{4}^{\left(n\right)}\\ r_{3n}=\left(c_{13}+c_{35}\alpha_{n}\right)A_{1}^{\left(n\right)}+\left(c_{36}+c_{34}\alpha_{n}\right)A_{2}^{\left(n\right)}+\left(c_{35}+c_{33}\alpha_{n}\right)A_{3}^{\left(n\right)}+\left(e_{13}+e_{33}\alpha_{n}\right)A_{4}^{\left(n\right)}\\ r_{4n}=\left(e_{31}+e_{35}\alpha_{n}\right)A_{1}^{\left(n\right)}+\left(e_{36}+e_{34}\alpha_{n}\right)A_{2}^{\left(n\right)}+\left(e_{35}+e_{33}\alpha_{n}\right)A_{3}^{\left(n\right)}+\left(\varepsilon_{31}+\varepsilon_{33}\alpha_{n}+j\varepsilon_{0}\right)A_{\left(4\right)}^{\left(n\right)} \end{array},n=1,2,3,4$$

Here, the *ε*_0_ is the dielectric constant in vacuum. Usually, after solving Equation (8), the *C*(*m*) can be determined. However, due to the inhomogeneity of Equation (8), the *C*(*m*) is difficult to extract directly. So, referring to the surface effective permittivity method [12], the acoustic field in the piezoelectric substrate can be easily described. The surface effective permittivity constant *ε_s_*(*s*) of the piezoelectric substrate is represented as:

(9) $$\varepsilon_{s}\left(s\right)=\frac{\bar{\sigma }\left(s\right)}{2\pi \left|s\right|\bar{\phi }{\left(s,x_{3}\right)|}_{x_{3}=0}}$$

where, *ϕ̄*(*s*, *x*_3_) is the electrical potential distribution in slowness domain of the piezoelectric substrate. *s* is the slowness, and *s* =1/*V_s_*.

Then, the weight factors *C*(*m*) can be deduced as following by the surface effective permittivity:

$$C_{n}=\frac{\Delta_{4n}}{\Delta_{0}}\frac{\bar{\sigma }(s)}{j2\pi fs}$$

thus, the effective permittivity constant *ε_s_*(*s*) was obtained by:

(10) $$\varepsilon_{s}\left(s\right)=\frac{\mathrm{js}}{\left|s\right|}\frac{\Delta_{0}}{\sum_{n=1}^{4}\Delta_{4n}A_{4}^{\left(n\right)}}$$

where Δ_0_ is the determinant value of the matrix in left side of Equation (8), Δ_4n_ is the algebraic complement of matrix elements *r*_4n_ in the matrix of [*r*_in_]. The *ε_s_* values in case various slowness *s* and normalized rotation (Ω*_i_*/ω, *i* = 1, 2, 3) can be extract. Hereof, after the analysis from the *ε_s_* curves in case the slowness, the SAW velocity can be determined under different rotation. Moreover, the gyroscope gain factor β relating to the frequency sensitivity of the gyroscope can also be determined by:

(11) $$\Delta f_{i}/f_{0}\approx \Delta V_{i}/V_{s}=\beta_{i}\times \Omega_{i}/\omega ,i=1,2,3$$

here, Δ*f* and Δ*V* are the frequency and velocity shifts due to the normalized rotation Ω_i_/ω.

### 2.3. Numerical Results and Discussion

As a numerical example, the gyroscopic effect of the SAW propagating along some common piezoelectric substrates like ST-X quartz, 128°YX LiNbO_3_ and X-112°Y LiTaO_3_ was considered. The mechanical parameters like the density, elastic constants, piezoelectricity and dielectric constants of the above mediums are listed in Table 1 [16].

**Figure 3.**
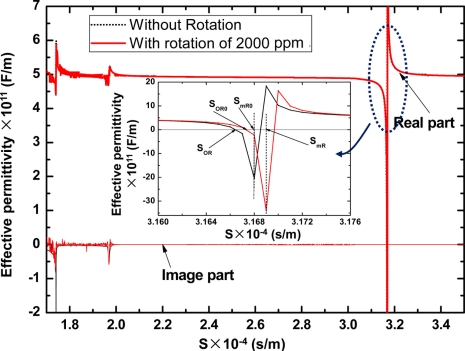
The surface effective permittivity curve on X-112°Y LiTaO_3_ with and without rotation.

Using Equations (8–10), the surface effective permittivity constant *ε_s_*(*s*) functioned as slowness (*s*) on the X-112°Y LiTaO_3_ substrate in the case of a normalized rotation of 2,000 ppm (Ω/*ω*) around the *x*_1_-axis was depicted as shown in Figure 3. From the picture, we can find that there are several discontinuous points in the curve of the real part of *ε_s_*(*s*), indicating different acoustic wave modes. The Rayleigh wave mode occurs at lower acoustic wave velocity (slowness of ∼3.2 × 10^−4^ s/m), and the image part of *ε_s_*(*s*) was equal to zero due to non-dissipation in acoustic energy. Meanwhile, there are pairs of zero point and pole points in the regime of Rayleigh wave mode. Obviously, the value of |*ε_s_*(*S_mR_*)| tends to infinity at the pole point of *S_mR_*, corresponding the situation of a metallic surface with limited charge distribution and zero surface potential. Thus, the *S_mR_* represents the slowness of Rayleigh wave propagating along the metallic surface with no attenuation. Additionally, the value of *ε_s_*(*S_oR_*) was equal to zero at the zero point of *S_oR_*, which chracterizes the situation of a free surface with limited electric potential and zero charge distribution, and it describes the Rayleigh wave propagating along the free surface with no attenuation. Usually, the phase velocity of the Rayleigh SAW under given rotation, *V_s_*, can be described by:

(12) $$V_{s}=\left(1/S_{\mathrm{OR}}+1/S_{mR}\right)/2$$

Thus, the velocity shift induced by the external rotation Δ*V_s_* is represented by:

(13) $${\Delta V}_{s}=V_{s^{-}}V_{0,}V_{0}=\left(1/S_{\mathrm{OR}0}+1/S_{mR0}\right)/2$$

here, the S*_OR0_* and *S_mR0_* are the slowness under no applied rotation. And then, the gyroscopic gain constant *β* can also be deduced by Equation (11).

Using the surface effective permittivity method described as above and the corresponding mechanical parameters listed in Table 1, the gyroscopic gain constant *β* and expected sensitivity were calculated and are listed in Table 2. From the calculated results, we find that the calculated *β* values agree well with the values reported in previous literatures [6–8], it indicates the validity of surface effective permittivity constant method. Also, from the calculated results, larger sensor response will be expected from the rotated X-112°Y LiTaO_3_ piezoelectric substrate.

## 3. Technical Realization

In this section, the fabrication process of the SAW sensor is described. It was composed of two delay lines with opposite direction on a same chip, and the corresponding oscillation circuit.

### 3.1. SAW Delay Lines

**Figure 4.**
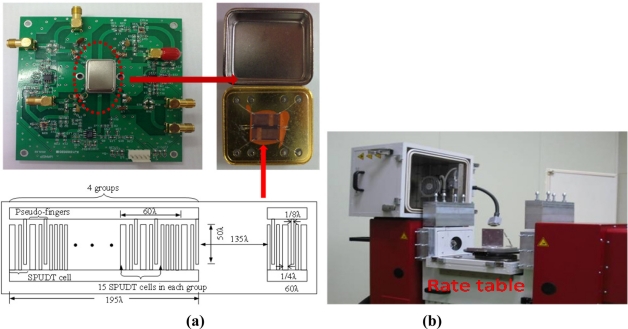
The PCB packaged with SAW gyroscope **(a)** and the precise rate table **(b)**.

EWC/SPUDT and a combed transducer were utilized to structure the SAW delay lines on X-112°Y LiTaO_3_ substrate to improve the frequency stability of the oscillator. The SAW velocity on the X-112°Y LiTaO_3_ substrate with 110 nm Al metallization was 3,295 m/s. The operation frequency of the SAW delay line is specifically given by 160 MHz, thus, the wavelength λ of the SAW is given by 20.6 μm. Two delay line patterns with opposite direction were fabricated on a same LiTaO_3_ wafer by standard photolithography techniques. Each delay line consists of a launching transducer and readout transducers; the length of the launching transducer was set to 195λ with four groups, which was about 80% of the center-to-center distance between the launching and readout transducers. The distance between the transducers was 135λ. In order to limit the total number of Al fingers in each transducer to about 60, the launching transducer was thinned into a comb structure. To keep the uniformity of the acoustic velocity, many pseudo-fingers are distributed between the SPUDT cells of the comb transducer. It means each group was composed of 15 SPUDT cells and some pseudo-fingers with length of 45λ, as shown in Figure 4(a). A large aperture of ∼1 mm (50λ) was used. The fabricated dual delay line is shown in Figure 4(a).

**Figure 5.**
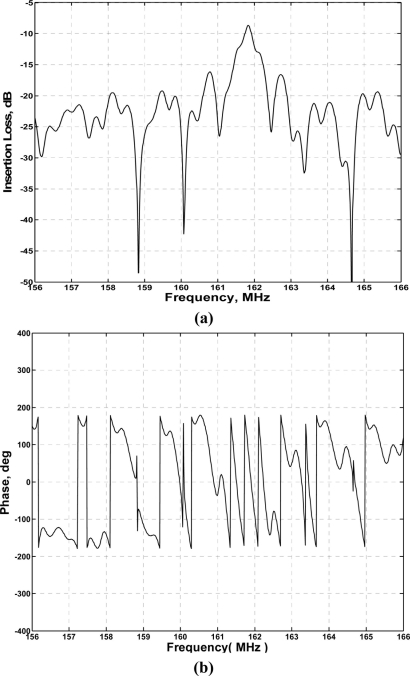
The measured amplitude **(a)** and phase **(b)** response of the fabricated SAW device.

Using the HP 8753D network analyzer, the amplitude and phase response of the SAW delay lines were measured under matched conditions, as shown in Figure 5. Low insertion loss of 9 dB and linearity phase response in 3-dB frequency bandwidth were observed.

### 3.2. SAW Oscillators

**Figure 6.**
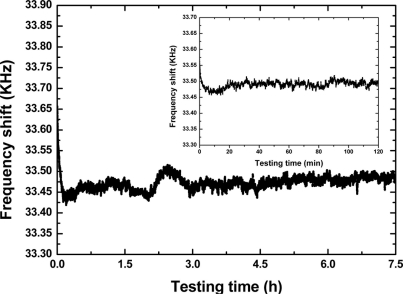
The measured long-term frequency stability of the fabricated SAW oscillator, insert: short-term frequency stability testing.

Next, the fabricated SAW device chip was loaded into a standard metal base, as shown in Figure 4(a). As the feedback of oscillators, the launching and read transducers of the fabricated SAW delay lines were connected by an oscillator circuit which was made of discrete elements (amplifier with a gain of 25 dB, phase shifter, mixer and LPF and so on) on a printed circuit board (PCB) as shown in Figure 4(a). The output of the amplifier was mixed in order to obtain a difference frequency in the MHz range. This technique allows us to double the detection sensitivity and reduce the influence of the thermal expansion of the substrate. The output of the oscillator was monitored and recorded by an oscilloscope and a programmable frequency counter. It is well-known that the frequency stability of the oscillator affects the threshold limit of detection and stability of the sensor directly. Thus, an experiment was performed to evaluate the frequency stability of the fabricated SAW oscillator using the programmable frequency counter under room temperature (20 °C). The oscillation was also modulated at the frequency point with lowest insertion loss by a strategically phase modulation. The measured frequency stability is shown in Figure 6. The shorted frequency stability (min) is up to 0.05 ppm as shown in the insert of Figure 6. The typical long-term stability (*h*) of the oscillator was measured as 0.4 ppm (Figure 6). From the measured results, excellent frequency stability was observed from the fabricated oscillator.

## 4. Sensor Experiments

**Figure 7.**
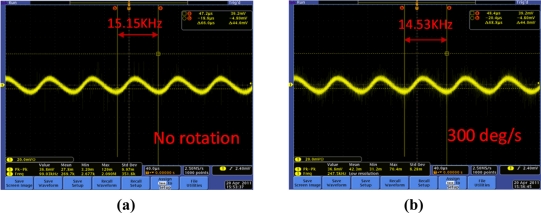
SAW gyroscope signal output depending on the different rotations **(a)** under no rotation and **(b)** under 300 deg/s rotation input.

**Figure 8.**
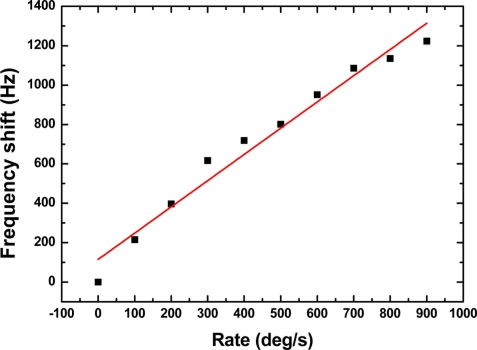
Sensitivity evaluation of the fabricated SAW gyroscope.

After mounting the SAW sensor onto the PCB board, the performance of the SAW rate sensor was evaluated. The experimental apparatus setup for performance evaluations was composed of a precision rate table [Figure 4(b)] and a universal oscilloscope. The PCB board with packaged SAW sensor was placed on the rate table in a vacuum chamber, which provides constant testing temperature and humidity to prevent any unwanted performance variations due to any temperature and humidity changes. An input voltage of ±5 V was applied to the PCB. The sensor output was monitored and recorded by the digital oscilloscope. When the sensor was subjected to external rotation, the sensor system frequency output signal changes linearly with the input rotation. Figure 7 shows the sensor response of the fabricated gyroscope towards external rotation of 300 deg s^−1^; a very clear frequency shift of 620 Hz was observed. The sensitivity and linearity of the present SAW gyroscope with rotation in the *x*-axis were evaluated as 1.332 Hz deg^−1^ s and 0.967, respectively, as shown in Figure 8. The measured sensitivity is ∼3 times larger than that of a reported similar gyroscope [8].

## 5. Conclusions

The SAW gyroscopic effect was analyzed theoretical by solving the piezoelectric medium equations of motion and the surface effective permittivity method. The calculated results indicate that among the common piezoelectric substrates a larger sensor response was observed from the rotated X-112°Y LiTaO_3_. Based on the theoretical analysis, a new SAW gyroscope on X-112°Y LiTaO_3_ substrate with operation frequency of 160 MHz was developed, \composed of two SAW delay line oscillators with opposite direction. Using a precise rate table, the sensor performance was evaluated experimentally. Sensitivity of 1.332 Hz deg^−1^ s over a large dynamic testing range (over 900 deg s^−1^), and good linearity were obtained.

## Figures and Tables

**Table 1.** The mechanical parameters of some common substrates.

**Materials**	**Euler angle**	**Density (kg/m^3^)**	**Stiffness (10N/m^2^)**	**Piezoelectricity (C/m^2^)**	**Dielectric Constants (×ε_0_)**
Quartz	ST-X (0°,132.75°,0°)	2,651	c_11_:8.674; c_33_:10.72;c_44_:5.794; c_12_:0.699; c_13_:1.191; c_14_:−1.791	e_11_:0.171; e_14_:−0.0436; e_36_:0.14	ε_11_:4.5; ε_33_: 4.6
	ST-X (0°,132.75°,33.3°)				
128°YX LiNbO_3_	(0°,37.86°,0°)	4,700	c_11_:20.3;c_33_:24.5;c_44_:6.0; c_12_:5.3; c_13_:7.5; c_14_:0.9	e_15_:3.7; e_22_: 2.5; e_31_: 0.2; e_33_: 1.3	ε_11_:44; ε_33_: 29
X-112°Y LiTaO_3_	(90°,90°,112.2°)	7,450	c_11_:23.3; c_33_:27.5; c_44_:9.4; c_12_:4.7; c_13_:8.0; c_14_:−1. 1	e_15_:2.6; e_22_: 1.6; e_31_: 0.0; e_33_: 1.9	ε_11_:41; ε_33_: 29

**Table 2.** The calculated gyroscopic effect for SAW along various substrates.

**Materials**	**Density (kg/m^3^)**	**Theoretical β and Expected Sensitivity (Hz deg^−1^ s)**	**Reported β in Previous Literatures**
ST-X quartz (Euler(0°,132.75°,0°))	2,651	−0.15/0.024 in *x*_2_-axis	−0.14 [9]
ST-X quartz (Euler (0°,132.75°,33.3°))	2,651	−0.19/0.03 in *x*_2_-axis	−0.73 [9]
128° YX LiNbO_3_ (Euler (0°, 37.86°, 0°))	4,700	−0.08/0.012 in *x*_2_-axis	−0.094 [7]
X-112° Y LiTaO_3_ (Euler(90°,90°,112.2°))	7,450	−0.38/0.06 in *x*_1_-axis	−0.39 [7]
